# Peroxisome proliferator-activated receptor alpha, PPARα, directly regulates transcription of cytochrome P450 CYP2C8

**DOI:** 10.3389/fphar.2015.00261

**Published:** 2015-11-04

**Authors:** Maria Thomas, Stefan Winter, Britta Klumpp, Miia Turpeinen, Kathrin Klein, Matthias Schwab, Ulrich M. Zanger

**Affiliations:** ^1^Dr. Margarete Fischer-Bosch-Institute of Clinical PharmacologyStuttgart, Germany; ^2^University of TuebingenTuebingen, Germany; ^3^Department of Clinical Pharmacology, University Hospital TuebingenTuebingen, Germany

**Keywords:** chromatin immunoprecipitation, diet–drug interaction, drug metabolism, genotype–phenotype correlation, HepaRG cells, nuclear receptors, transcriptional regulation, WNT/β-catenin

## Abstract

The cytochrome P450, CYP2C8, metabolizes more than 60 clinically used drugs as well as endogenous substances including retinoic acid and arachidonic acid. However, predictive factors for interindividual variability in the efficacy and toxicity of CYP2C8 drug substrates are essentially lacking. Recently we demonstrated that peroxisome proliferator-activated receptor alpha (PPARα), a nuclear receptor primarily involved in control of lipid and energy homeostasis directly regulates the transcription of CYP3A4. Here we investigated the potential regulation of CYP2C8 by PPARα. Two linked intronic SNPs in PPARα (rs4253728, rs4823613) previously associated with hepatic CYP3A4 status showed significant association with CYP2C8 protein level in human liver samples (*N* = 150). Furthermore, siRNA-mediated knock-down of PPARα in HepaRG human hepatocyte cells resulted in up to ∼60 and ∼50% downregulation of CYP2C8 mRNA and activity, while treatment with the PPARα agonist WY14,643 lead to an induction by >150 and >100%, respectively. Using chromatin immunoprecipitation scanning assay we identified a specific upstream gene region that is occupied *in vivo* by PPARα. Electromobility shift assay demonstrated direct binding of PPARα to a DR-1 motif located at positions –2762/–2775 bp upstream of the CYP2C8 transcription start site. We further validated the functional activity of this element using luciferase reporter gene assays in HuH7 cells. Moreover, based on our previous studies we demonstrated that WNT/β-catenin acts as a functional inhibitor of PPARα-mediated inducibility of CYP2C8 expression. In conclusion, our data suggest direct involvement of PPARα in both constitutive and inducible regulation of CYP2C8 expression in human liver, which is further modulated by WNT/β-catenin pathway. *PPARA* gene polymorphism could have a modest influence on CYP2C8 phenotype.

## Introduction

The human CYP2C subfamily consists of four highly homologous genes, CYP2C18, CYP2C19, CYP2C9, and CYP2C8, which are localized in this order in a ∼390 kb gene cluster on chromosome 10q23.3. The CYP2C8 is responsible for the oxidative metabolism of many clinically available drugs from a diverse number of drug classes, including thiazolidinedione and meglitinide antidiabetics, non-steroidal anti-inflammatory agents (NSAIDs), antimalarials (e.g., amodiaquine, chloroquine), chemotherapeutics (e.g., taxanes, imatinib), and numerous other drugs (Daily and Aquilante, 2009; Xiaoping et al., 2013; Zanger and Schwab, 2013). CYP2C8 is also involved in the endogenous metabolism of arachidonic acid and all-*trans*-retinoic acid to epoxyeicosatrienoic acids and 4-hydroxy-all-*trans*-retinoic acid, respectively (Zeldin et al., 1996; McSorley and Daly, 2000). CYP2C8 shares more common substrates with CYP3A4 than it does with CYP2C9, despite its closer sequence homology to CYP2C9 (Totah and Rettie, 2005). All CYP2C enzymes are primarily expressed in liver although lower levels of functional CYP2C enzymes are also expressed in extrahepatic tissues, e.g., in human small intestine and in cardiovascular tissues (DeLozier et al., 2007; Achour et al., 2014). While CYP2C9 is the major CYP2C subfamily isoform in human liver, CYP2C8 has been suggested to be the major fetal CYP2C form (Achour et al., 2014; Johansson et al., 2014). Interestingly, hepatic expression of CYP2C8 is rather strongly correlated to CYP3A4 (Naraharisetti et al., 2010; Achour et al., 2014).

Although the CYP2C subfamily members CYP2C9 and CYP2C19 show clinically relevant genetic polymorphism, there is conflicting data regarding polymorphic effects on CYP2C8. While it has been reported that clearance of CYP2C8 substrates repaglinide, rosiglitazone, and pioglitazone is increased in homozygous and heterozygous carriers of *CYP2C8^∗^3* (Niemi et al., 2005; Kirchheiner et al., 2006; Tornio et al., 2008), other *in vitro* and *in vivo* showed contradictory results (Bahadur et al., 2002; Dai et al., 2001; Daily and Aquilante, 2009). Moreover, the CYP2C8^∗^4 allele did not influence the pharmacokinetics of repaglinide (Niemi et al., 2003).

Thus, compared to other CYP2C genes, CYP2C8 appears to be less strongly affected by genetic variation and consequently regulatory events may have a more significant impact on variability. The transcriptional regulation of CYP2C genes has been thoroughly studied implying constitutive regulation by involving the liver-enriched receptor HNF4 (Jover et al., 2001; Ferguson et al., 2005; Rana et al., 2010; Yue et al., 2010) as well as inducible regulation with xenobiotic-sensing receptors CAR, PXR, and glucocorticoid receptor (GR) playing major roles (Pascussi et al., 2000; Ferguson et al., 2005; Chen and Goldstein, 2009; Rana et al., 2010). Interestingly, Prueksaritanont et al. (2005) observed pronounced induction of CYP3A4 and CYP2C8 in human hepatocytes by a series of fibrates including clofibric and fenofibric acids and gemfibrozil, but failed to link this to the fibrate receptor, PPARα. The finding was confirmed by other studies and appeared to be human-specific (Richert et al., 2008; Rakhshandehroo et al., 2009). While PPARα had been shown to transcriptionally activate some Phase II conjugating enzymes (e.g., EPHX2, GSTA, and UGT1A9; Barbier et al., 2009), direct regulation of cytochrome P450s was only recently shown by our group (Klein et al., 2012; Thomas et al., 2013). Elucidation of the molecular mechanism of PPARα-mediated regulation of CYP3A4 revealed direct transcriptional activation of the CYP3A4 promoter via at least three functional PPARα-binding regions (PBR-I, -II, and -III) within ∼12 kb of the CYP3A4 upstream gene region (Thomas et al., 2013). More recently, we found that the PPARα-mediated effects on CYP expression were additionally modulated by the WNT/β-catenin pathway (Thomas et al., 2015b).

In this context of *CYP2C8* pharmacogenetics and expression regulation, the aims of this study were: (a) to characterize hepatic CYP2C8 expression variability in 150 liver samples from white individuals; (b) to assess the impact of two *PPARA* polymorphisms, previously shown to correlate with CYP3A4, on the expression and activity of CYP2C8; (c) to investigate the potential direct regulation of CYP2C8 by PPARα in human hepatocytes; and (d) to further elucidate the molecular basis for the modulation of PPARα-mediated effects on CYP2C8 by the WNT/β-catenin pathway. We demonstrate that PPARα directly binds and regulates CYP2C8 via specific binding elements within the *CYP2C8* promoter. We also find a moderate influence of *PPARA* gene polymorphisms on hepatic CYP2C8 phenotype. These novel findings may help to better understand the interindividual variability in the response to various CYP2C8 drug substrates.

## Materials and Methods

### Cell Culture and Treatments

Detailed description of culturing HepaRG cells can be found elsewhere (Klein et al., 2015). Briefly, HepaRG cells (batch HPR101007) were obtained from Biopredic International (Rennes, France) and expanded according to the provider’s instructions. The cells were cultivated for the first 14 days in HepaRG growth medium based on William’s E Medium with supplements. At the final stage, HepaRG cells reached a differentiated hepatocyte-like morphology and showed liver-specific functions. The cells were further maintained in HepaRG differentiation medium for the duration of the experiments with exchange of medium every 2 days. All cells were maintained at 37°C and 5% CO_2_ in a humidified atmosphere throughout the experiment.

### Transfections with siRNAs

For the RNA interference experiments, HepaRG cells were transfected with 20 nM siRNAs using 10 pmol Lipofectamine RNAiMAX Transfection Reagent (Life Technologies) in 12-well plates with serum-free medium. The siRNA targeting PPARα (Thomas et al., 2014, 2015a), β-catenin (Thomas et al., 2015b), and a non-targeting siRNA as a negative control (Lo GC Duplex 2) were obtained from Life Technologies. One-hundred microliters of the transfection cocktail was added per well to the cells containing 100 μl culture medium. Upon 20 min of complex formation, the liposomes were given to the cells. Twenty-four hours after the transfection cells were treated for an additional 48 h with 100 μM of PPARa agonist, WY14,643 (Sigma–Aldrich) or solvent control, DMSO (Sigma–Aldrich).

### Human Liver Cohort

Liver tissues and corresponding blood samples were previously collected from 150 patients of Caucasian ethnicity (71 males and 79 females; average age of the subjects 58 ± 14 years). Patients who suffered from hepatitis, cirrhosis, or alcohol abuse were excluded. All tissue samples had been examined by a pathologist and only histologically non-tumorous tissue was used (Schröder et al., 2013). The study was approved by the ethics committees of the medical faculties of the Charité, Humboldt University, and of the University of Tuebingen and conducted in accordance with the Declaration of Helsinki. Written informed consent was obtained from each patient.

### Quantitative Real-time RT-PCR Gene Expression Analysis

For the determination of the absolute amounts of CYP2C8 mRNA expresion in the cohort of the liver samples, high quality total RNA was isolated from liver tissue using Trizol/Qiagen RNeasy protocol as described previously (Gomes et al., 2009). Synthesis of cDNA was performed with 1 μg RNA using the TaqMan Reverse Transcription Kit (Applied Biosystems) according to the supplier’s instructions. Expression of CYP2C8 mRNA in liver tissue was performed using a commercially purchased gene expression assay (Applied Biosystems, Hs_00946140_g1) with a TaqMan 7500 system (Applied Biosystems). A standard curve was obtained using CYP2C8 cDNA-containing linearized plasmid DNA purchased from OriGene (SC107944). Raw data were normalized to RPLP0 (60S large ribosomal protein P0) expression which was shown to be the most suitable reference gene in the liver tissue cohort using geNorm analysis (Vandesompele et al., 2002). RPLP0 was determined in the same samples using the endogenous control assay (4326314E) from Applied Biosystems. Normalized values were adjusted to the median value of all samples.

For qRT-PCR analysis of treated HepaRG cells, total RNA was isolated from HepaRG cells using the RNeasy Mini Kit, including on-column genomic DNA digestion with RNase free DNase Set (Qiagen). RNA was reverse transcribed to cDNA with TaqMan Reverse Transcription Reagents (Applera GmbH). Quantification of CYP2C8 expression was performed using ABI Applied Biosystems^^®^^ 7500 Real-Time PCR System following the manufacturer’s instructions using Life Technologies Assays (Hs_00946140_g1 for CYP2C8 and 4326314E for housekeeping gene, RPLP0). The mRNA expression levels were normalized to the RPLP0 mRNA expression. Relative gene expression changes were calculated using the delta delta Ct (ΔΔCt)-method (Livak and Schmittgen, 2001; Feuer et al., 2015).

### Western Blot Detection of Protein Expression

CYP2C8 protein expression in the liver microsomes of the liver samples cohort or following HepaRG cell treatments was analyzed by Western blot. Ten micrograms of protein were separated by electrophoresis on a 10% SDS-polyacrylamide gel and blotted to nitrocellulose membranes. Anti-human CYP2C8 monoclonal antibody (Rabbit anti-human CYP2C8, Puracyp # Hu-A004) and IRD800-labeled secondary anti-rabbit antibody (Li-cor) were used for detection with an Odyssey system (Li-cor). For absolute quantification, a standard curve was generated by coanalyzing 250–4000 fmol of recombinantly expressed CYP2C8 (Becton Dickinson Gentest 455112) in each experiment.

### Transfections and Luciferase Reporter Analyses

Cells were transfected with the Firefly luciferase reporter constructs using standard methods as recently described (Thomas et al., 2013, 2015a). The plasmid pRL-CMV, encoding Renilla luciferase under the control of a constitutively active viral promoter, was co-transfected for normalization purposes. 24 h after seeding of the cells, 800 ng of plasmid DNA (750 ng of the respective Firefly luciferase reporter plasmid, 50 ng pRL-CMV) were transfected per well of a 24-well plate using Lipofectamine 2000 (Invitrogen). Firefly luciferase reporter plasmids used in the study were: a pGL3-based 4xPPRE-driven reporter for luciferase expression under the control of 4 rat PPRE sites responsive to activation by the PPARα (“pGL3-PPRE pos”; Thomas et al., 2013) and a pGL3-based reporter plasmids driven by approximately 1000 bp of the human CYP2C8 promoter region between –2500/–3500 bp (“pGL3-CYP2C8-Site C”) and –8500/–9500 bp (“pGL3-CYP2C8-Site A”) to test the functional activation of the predicted sites. For the confirmations of the functional activity of the binding motifs following mutations were introduced (shown in bold): *TC***CCA***TTTGATG***CTT***C* [“pGL3-CYP2C8-Site A^∗^(mut)”]; *A***TC***TCGAAGT***CT***AC* [“pGL3-CYP2C8-Site C^∗^ (mut)”]. The plasmids were generated by GenScript sequence Synthesis Company. Transfection experiments with the pGL3Basic empty vector were conducted as controls. Cells were incubated with 100 μM WY14,643 or v/v DMSO control for 48 h prior to lysis with 1x Passive Lysis Buffer (Promega) and luciferase activity determination as previously described (Thomas et al., 2013).

### Assessment of CYP Metabolic Activities

Cytochrome P450 enzyme activities were determined in the cohort of human liver samples and HepaRG cell culture supernatants using a liquid chromatography with tandem mass spectrometry-based substrate cocktail assay, as previously described (Feidt et al., 2010). The CYP substrate mix was added to cell cultures after 45 h of incubation with the enzyme inducers as detailed above. The following substrates were used: 50 μM phenacetin (CYP1A2), 25 μM bupropion (CYP2B6), 5 μM amodiaquin (CYP2C8), 100 μM tolbutamide (CYP2C9), 5 μM propafenone (CYP2D6), 100 μM atorvastatin (CYP3A4). Aliquots of the supernatant were taken after 3 h of incubation at 37°C. Metabolite formation was normalized to cellular protein content.

### Fluorescence-based Electromobility Shift Assays

Human PPARα and RXRα proteins were synthesized using expression plasmids and TNT T7 Quick Coupled Transcription/Translation System (Promega). EMSA probes consisted of IRD700-fluorescence-labeled double-stranded oligonucleotides prepared by hybridization of complementary single-stranded 5′IRD700 labeled oligonucleotides (Metabion) by annealing at 100°C for 3 min and gradually cooling down at 0.1°C/sec to 20°C. The reaction mixtures (20 μl) containing 3 μl of TNT-Protein, 50 fmol of fluorescence-labeled probe, 1 μg of poly(dI-dT), 10 mM Tris-HCl, (pH 7.5), 50 mM KCl, 10 mM MgCl2, 3.5 mM DTT, 0.25% Tween 20 were incubated for 30 min at room temperature. The reaction mixtures were then loaded on a native pre-electrophoresed 5% acrylamide gel (acrylamide/bis 30:1) and run at 100 V for 1.5 h in TGE-Buffer (25 mM Tris, 0.19 M Glycine, 1.34 mM EDTA, pH 8.3). Fluorescence was detected using Odyssey Infrared scanner (Li-cor).

The oligonucleotides used as fluorescent labeled probes for EMSA are listed in the Supplementary Table S1. Anti–PPARα antibody (sc-9000; Santa Cruz Biotechnology) was used for the supershift assays.

### Chromatin Immunoprecipitation Assay

Chromatin immunoprecipitation was performed using MAGnify Chromatin Immunoprecipitation Kit (Invitrogen) according to the manufacturer’s description and as previously described (Thomas et al., 2013). Briefly, DNA was sheared by sonication to an average length of ∼800 bp using Bioruptor (Diagenode) and incubated with 10 μg of anti-PPARα antibody (PP-H0723-00; R&D Systems), previously bound to 10 μl of magnetic beads at 4°C for 2 h, and DNA was purified using DNA purification beads and eluted in 150 μl of elution buffer. Promoter occupation was analyzed with 5 μl of immunoprecipitated DNA by Sybr-Green polymerase chain reaction (PCR). The results were normalized to HMGCR (3-hydroxy-3-methyl-glutaryl-CoA reductase) promoter region (–21970/–22200 bp) used as positive control (van der Meer et al., 2010) and the untranscribed region Untr-5 as negative control (Hariparsad et al., 2009).

### Statistical Analyses

Statistical analyses were performed using software R-3.2.0^1^ with additional packages coin_1.0-24 (Hothorn et al., 2006), quantreg_5.11^2^, and RVAideMemoire_0.9-50^3^. Spearman’s rank correlation coefficient was used to assess associations between CYP2C8 phenotypes (amodiaquine *N*-demethylation, CYP2C8 protein and mRNA expression) and of PPARα protein and mRNA expression. The effect of each PPARA-SNP on each of the three CYP2C8 phenotypes was studied in three genetic models: dominant, recessive, and additive model. For the first two genetic models, Wilcoxon–Mann–Whitney tests were applied for univariate association analyses, whereas the last model was investigated by Spearman correlation tests. In addition, multivariate analyses were performed considering 10 non-genetic factors (age, gender, nicotine and alcohol intake, exposure to P450 inducers, total bilirubin, GGT, CRP, cholestasis, diagnosis; Klein et al., 2010). To be more precise, for each of the CYP2C8 phenotypes, each SNP, and each genetic model, two median regression fits were compared with function anova.rq of library quantreg (using a rank test with Wilcoxon scores): (a) with the SNP in the respective genetic model plus the ten non-genetic factors and (b) only with the ten non-genetic factors. Reported *p*-values were adjusted for multiple testing (Bonferroni) where appropriate. For all calculations, all data were used, including all outlier data presented in various graphics. All statistical tests were two-sided and statistical significance was defined as *p* < 0.05; 95% confidence intervals (95% CI) were reported where appropriate.

## Results

### Population Variability of Hepatic CYP2C8

**FIGURE 1:**
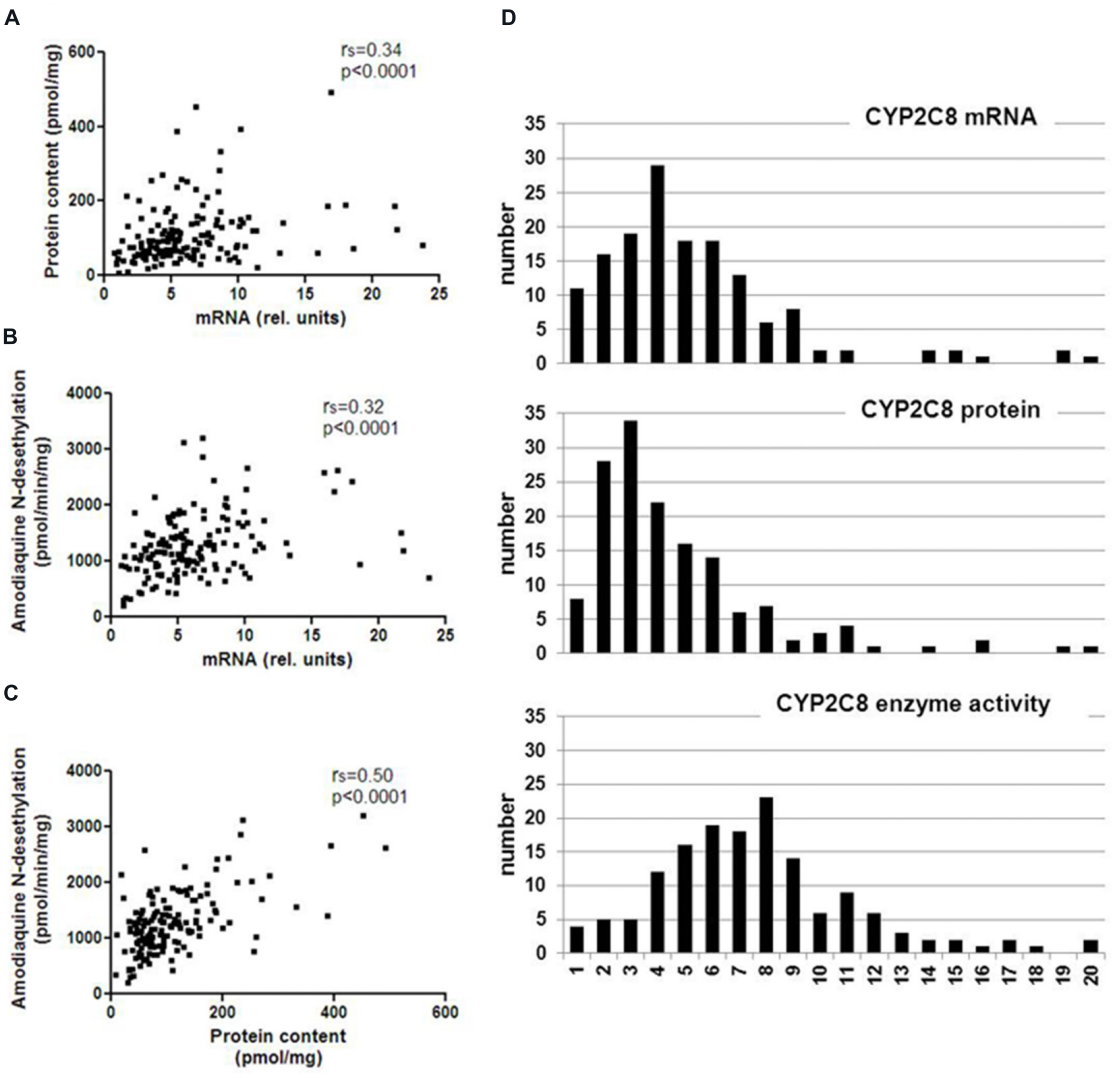
**Spearman correlations and population distribution of hepatic CYP2C8 phenotypes.** CYP2C8 mRNA was determined by a specific TaqMan real-time RT-PCR assay, protein was determined by Western blotting and enzyme activity was measured by LC–MSMS analysis of amodiaquine *N*-desethylation in human liver microsomes (*n* = 150). Results are means of duplicate measurements. **(A–C)** Spearman’s rank correlation coefficients are indicated as *r*_s_ and statistical significance for all comparisons was *p* < 0.0001. **(D)** Histograms showing population distributions for mRNA (top), protein (middle), and enzyme activity (bottom) using 20 bins over the entire phenotype range.

We quantitated CYP2C8 hepatic phenotypes, i.e., mRNA, protein, and enzyme activity in RNA and microsomes of a cohort of 150 human liver samples, and examined the effect of non-genetic parameters. CYP2C8 expression varied considerably at the different phenotype levels and was not normally distributed (**Table 1**, **Figure 1**). Fold-variation was highest (65.5-fold) for protein and lowest (15.7-fold) for enzyme activity (**Table 1**), while the coefficient of variation (cv) as a normalized measure of variability was more comparable between the different phenotypes. These values are comparable to previous studies (Rodríguez-Antona et al., 2007; Naraharisetti et al., 2010), however, compared to our studies on other liver drug metabolizing enzymes [e.g., CYPs 2D6 (Zanger et al., 2001), 2B6 (Hofmann et al., 2008), and 3A4 (Wolbold et al., 2003)], CYP2C8 variation appeared to be somewhat less pronounced. All three CYP2C8 phenotypes were significantly, but moderately correlated to each other (**Figure 1**). We also confirmed a significant correlation between CYP2C8 and CYP3A4 (e.g., *r*_s_ = 0.51, *P* < 0.0001 for protein level; Naraharisetti et al., 2010; Achour et al., 2014).

**Table 1:** Population variability of hepatic CYP2C8 expression phenotypes (*n* = 150).

	CYP2C8 mRNA	CYP2C8 Protein	CYP2C8 Activity
Minimum	0.08	7.52	204.2
Median	0.54	88.42	1212.6
Maximum	2.38	492.23	3212.2
Normal distribution	No	No	No
Ratio maximum/minimum	29.8	65.5	15.7
Coefficient of variation (-%)	65.46	74.11	43.19

### Non-genetic Factors Influencing CYP2C8 Expression

Available documentation to the liver donors included demographic, clinical, and self-reported data as previously reported (Klein et al., 2010). We used multivariate modeling to analyze whether any of these parameters had an impact on CYP2C8 phenotype. We identified cholestasis and increased bilirubin and GGT levels as factors being significantly associated with at least one microsomal CYP2C8 phenotype (**Table 2**). Liver donors diagnosed with cholestasis (Nies et al., 2009) had lower CYP2C8 protein levels (median difference, –26.8 pmol/mg [95% CI, 6.1–47.8]; donors with abnormal bilirubin serum levels had lower levels of protein (–36.47 pmol/mg [95% CI, 17.21–58.56]) and enzyme activity (–351.8 pmol/mg/min [95% CI, 125.95–562.92]); and donors with elevated serum gamma glutamyl transferase also had decreased protein levels (–17.52 pmol/mg [95% CI, 0.83–35.62]). After correction for multiple testing only increased serum bilirubin levels remained significantly associated with decreased CYP2C8 protein and enzyme activity.

**Table 2:** Influence of non-genetic factors on CYP2C8 phenotype.

Non-genetic factor^a^	Association test^b^	mRNA^c^	Protein^c^	Activity^c^
SEX	WMW	0.119	0.068	0.618
AGE	Spearman	0.321	0.577	0.106
NIC	WMW	0.375	0.988	0.895
ALC	WMW	0.257	0.681	0.149
DIAG	KW	0.363	0.198	0.231
CHOL	WMW	0.053	0.013	0.087
IND	WMW	0.488	0.729	0.552
BILI	WMW	0.094	**0.0004**	**0.003**
GGT	WMW	0.51	0.041	0.019
CRP	WMW	0.622	0.083	0.403

*^a^NIC, nicotine consumption: non-smokers vs. smokers (>1 cigarettes/day); ALC, alcohol consumption: none vs. >1 times/week; IND, exposure to known inducers of P450; BILI, serum total bilirubin; GGT, serum gamma glutamyl transferase; CRP, C-reactive protein; DIAG, diagnosis; CHOL, cholestasis.*

*^b^WMW, Wilcoxon–Mann–Whitney test; Spearman, Spearman’s correlation test; KW, Kruskal–Wallis test.*

*^c^p-values for univariate analyses using the given association test and were not corrected for multiple testing; p-values significant after Bonferroni correction are shown in bold type.*

### Hepatic Expression and Genotype of *PPARA* Correlates with CYP2C8 Phenotypes

**FIGURE 2:**
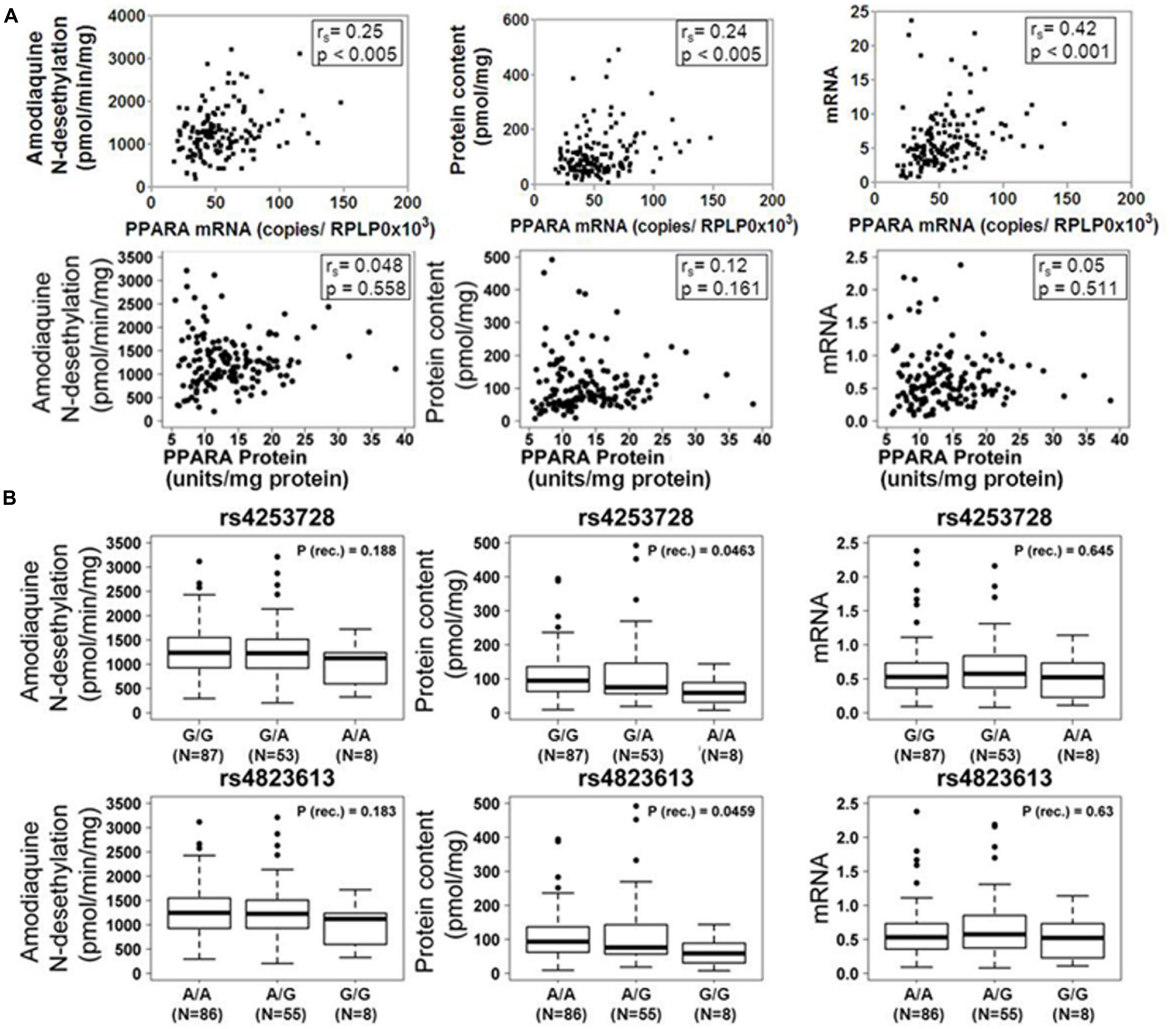
**Influence of PPARα expression and genotype on the CYP2C8 phenotype. (A)** Scatter plots of CYP2C8 phenotypes (amodiaquine N-desethylation, CYP2C8 protein and mRNA expression) vs. PPARα mRNA (top) and protein (bottom) expression. Spearman’s rank correlation coefficient *r*_s_ and corresponding *p*-values are given. **(B)** Box-and-whisker plots of CYP2C8 phenotypes (Amodiaquine *N*-desethylation, CYP2C8 protein and mRNA expression) for two previously described intronic PPARA variants. Genotypes are indicated as G/G, G/A, and A/A (for rs4253728) and A/A, A/G, and G/G (for rs4823613). P (rec.), unadjusted *p*-value of Wilcoxon–Mann–Whitney test for recessive genetic model; N, number of individuals per group; numbers for the three genotype groups do not add up to 150 due to missing values. Outliers were included in all calculations.

Based on our previous studies describing impact of PPARα on the regulation of P450 enzymes, we assessed the correlation between PPARα expression and CYP2C8 phenotypes in our human liver cohort. As shown in **Figure 2A**, mRNA expression of *PPARA* was moderately but significantly correlated to mRNA (*r*_s_ = 0.42; *p* < 0.0001), protein (*r*_s_ = 0.24; *p* < 0.005) and activity (*r*_s_ = 0.25; *p* < 0.005) of CYP2C8, while we did not observe any significant correlation between PPARα protein and any phenotype of CYP2C8.

Additionally, we assessed the impact of two linked *PPARA* variants, rs4253728 and rs4823613, previously shown to correlate with CYP3A4 (Klein et al., 2012), on the expression and activity of CYP2C8 by univariate analysis, applying three different genetic models (dominant, recessive, or additive, see Materials and Methods). The additive model is based on the assumption of a gene-dose effect, such that heterozygotes are phenotypically intermediate between homozygous wild-types and mutants. Without correction for non-genetic factors, we found for both SNPs significantly decreased levels of CYP2C8 protein expression in homozygous carriers of the minor allele (**Figure 2B**). However, after correction for non-genetic factors and adjustment for multiple testing these relationships did not remain significant.

### Ligand-mediated PPARα Activation and *PPARA* Gene Knockdown Modulates CYP2C8 Expression in HepaRG Cells

**FIGURE 3:**
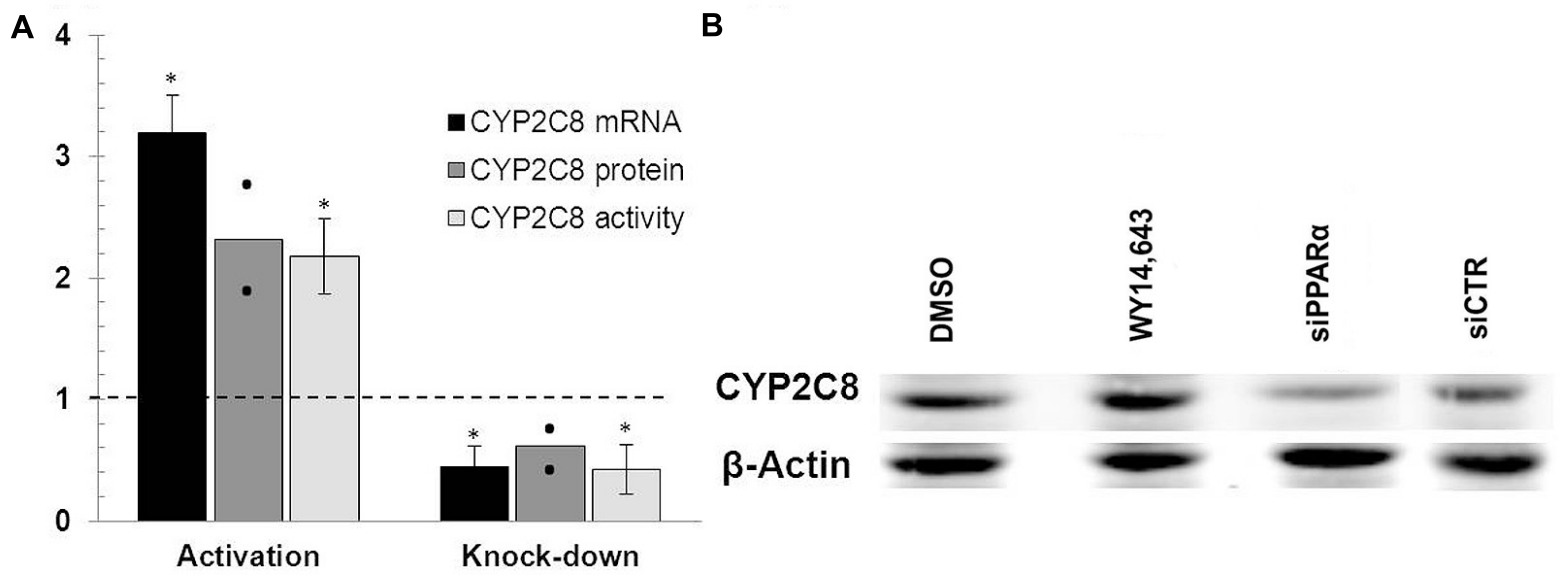
**PPARα knockdown and activation in HepaRG cells. (A)** mRNA levels were measured in HepaRG cultures cultured in three independent differentiation batches 72 h after PPARα activation using ligand WY14,643 (left part of the diagram) or siRNA-mediated PPARα knock-down (right part of the diagram) and compared with mRNA levels measured in cells treated with either DMSO (left) or non-targeting siRNA (right) set at 1.0. The graph shows the means from three (mRNA, activity) or two (protein) independent experiments, with error bars indicating standard deviations (mRNA, activity) and dots representing individual data points (protein). ^∗^, Statistically significant (*P* < 0.05, paired *t*-test). **(B)** Representative Western Blot analysis of the corresponding protein levels of CYP2C8 following either ligand-mediated induction (lanes DMSO and WY14,643) or siRNA-mediated knock-down (lanes siPPARa and siCTR) of PPARα.

To directly investigate the functional impact of PPARα on CYP2C8 we applied two available strategies, namely stimulation with the canonical PPARα ligand, WY14,643, and depletion of PPARα using siRNA-mediate gene knock down. As shown in **Figure 3A**, treatment of HepaRG cells with 100 μM WY14,643 significantly induced the expression of CYP2C8 at mRNA (more than threefold), protein (**Figure 3B**, lane WY14,643) and activity (over twofold) levels, confirming earlier observations by Prueksaritanont et al. (2005). In the same experimental set-up, transfection of HepaRG cells with PPARα-targeting siRNA resulted in >50% reduction in the expression of mRNA and >40% decrease in protein levels (**Figure 3B**, lane siPPARa) of CYP2C8 as compared with cells treated with non-silencing siRNA. The measurement of corresponding CYP2C8 enzyme activity after *PPARA* gene silencing resulted in an average amodiaquine *N*-desethylation reduction over 45% as compared with non-targeting control. These findings demonstrated that PPARα mediates both basal and inducible regulation of CYP2C8 in human hepatocytes.

### Chromatin Immunoprecipitation (ChIP) Identifies a CYP2C8 Promoter Region Occupied by PPARα in HepaRG Cells

**FIGURE 4:**
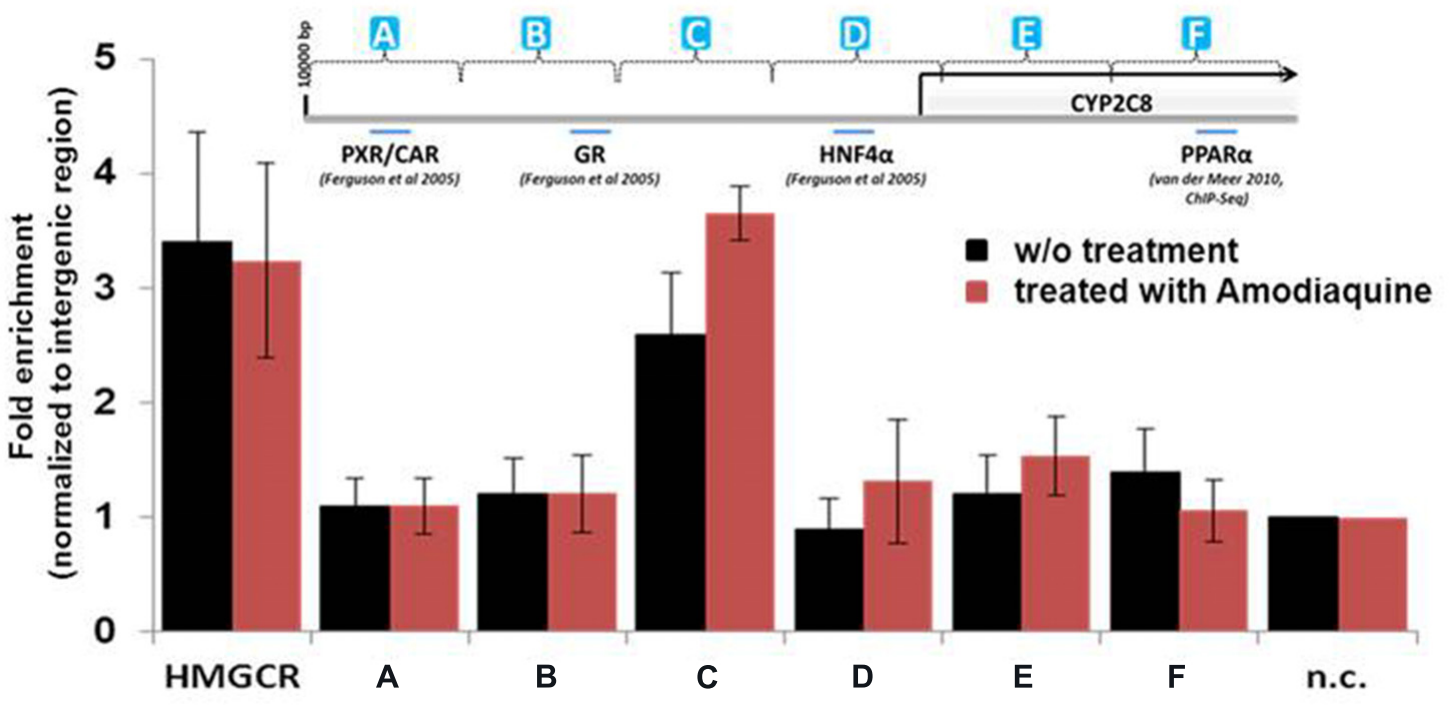
**Binding of PPARα to the CYP2C8 promoter *in vivo*.** Precipitated DNA from HepaRG cells without (w/o) treatment or after treatment with amodiaquine (10 μM for 6 h) was purified and was used, together with input DNA, as template for Sybr-Green PCR using a total of 12 primer pairs spanning approximately 10 kb of the CYP2C8 promoter region. Raw Ct (cycle threshold) values were normalized to input DNA to calculate the percentage of DNA immunoprecipitated. Primers encompassing the PPRE of the human HMGCR gene were used as positive control. Means relative to negative control primer pair (n.c.) are shown. (A–F) Schematic representation of CYP2C8 promoter regions, which were subjected for the ChIP analysis. Promoter scheme includes binding sites of previously described transcription factors.

*In silico* analysis of the CYP2C8 promoter and upstream region (10 kb) identified a number of putative direct repeat DR1 and DR2 motifs with different degrees of homology to the consensus peroxisome proliferator response element, PPRE, AGGTCA half site, although no 100% consensus motif was found. Thus, we systematically screened ∼10 kb of upstream region by ChIP of isolated chromatin from HepaRG cells using a total of 12 primer pairs. As evident from **Figure 4**, one region designated as PPARα-binding region (PBR-C, spanning between –2500 and –3300 bp) showed significant enrichment of promoter binding by PPARα compared with unoccupied intermediary gene regions and negative control (n.c.). Interestingly, pre-treatment of hepatocytes with 10 μM of CYP2C8 substrate, amodiaquine, resulted in ≈40% higher enrichment of PPARα occupation within this region (**Figure 4**, red bars).

### PPARα Directly Binds to the Specific Motifs of CYP2C8 Promoter and Activates its Expression.

**FIGURE 5:**
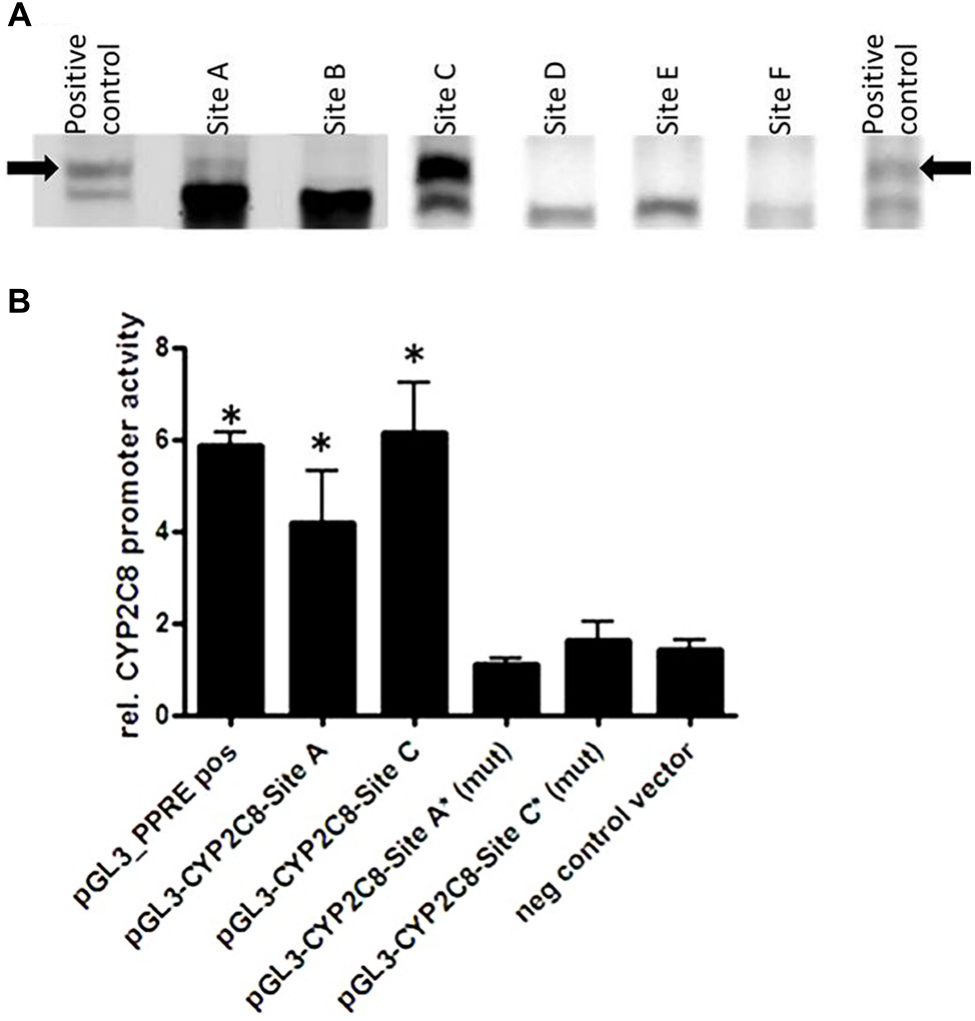
**Direct PPARα-mediated regulation of CYP2C8. (A)** Electrophoretic mobility shift assays using *in vitro* translated proteins was used for assessment the binding of fluorescence-labeled double-stranded oligonucleotide probes corresponding to the indicated regions within CYP2C8 promoter (selection out of 15 tested probes is shown). As a positive control for binding, the known PPARα/RXRα binding site of the rat ACOX1 gene was used (Thomas et al., 2013). Complexes of PPARα/RXRα heterodimers with the oligonucleotides are marked by an arrow. **(B)** Luciferase reporter gene constructs containing the sequences of the CYP2C8 promoter, PBR-A (–9500/–8500 bp) and PBR-C (–2500 to –3500 bp) were cotransfected with a PPARα expression plasmid and renilla-luciferase expression vector into HuH7 cells. Cells were treated with either 100 μM WY14,643 or vehicle DMSO for 48 h before measurement of firefly/renilla luciferase activities. Firefly luciferase activities were normalized to renilla luciferase activities to consider changes in the transfection variability and to DMSO control, set as 1. Data are means of three independent experiments, each performed in triplicates in 96-well format; (mut), mutated sites; ^∗^, statistically significant (*P* < 0.05) compared with DMSO treatment set to 1.

We next investigated whether PPARα/RXRα heterodimer can directly bind to their bioinformatically predicted potential sites using fluorescence-based EMSA. **Figure 5A** shows results for a selection of six motifs with the highest score for binding to the *in silico* predicted DR1/DR2 motifs out of a total of 15 tested motifs (Supplementary Table S1). We found that PPARα/RXRα specifically bound to two identified motifs, one of which is the DR-4 PXR/CAR-binding site, TCAACT**TTGA**TGACCC positioned between (–8806/–8822), previously identified by Ferguson et al. (2005). However, the newly identified DR1 PPRE motif, AGTTCG**A**AGTTCA within the identified PBR-C positioned between –2762/–2775 bp was bound with much higher apparent affinity (**Figure 5A**).

Furthermore, results of HuH7 cell cotransfection experiments performed in the absence and presence of WY14,643 are shown in **Figure 5B**. Luciferase reporter gene analysis of the CYP2C8 upstream promoter region incorporating PBRs- A and C showed over fourfold induction of PPARα activation by WY14,643. Furthermore, mutation of sites DR4-A and DR1-C completely abolished inducible PPARα-dependent transactivation of CYP2C8. These data further confirm functional activity of two motifs within CYP2C8 promoter, which can be bound by PPARα.

### WNT/β-Catenin Functions as an Inhibitor of PPARα-mediated CYP2C8 Induction

**FIGURE 6:**
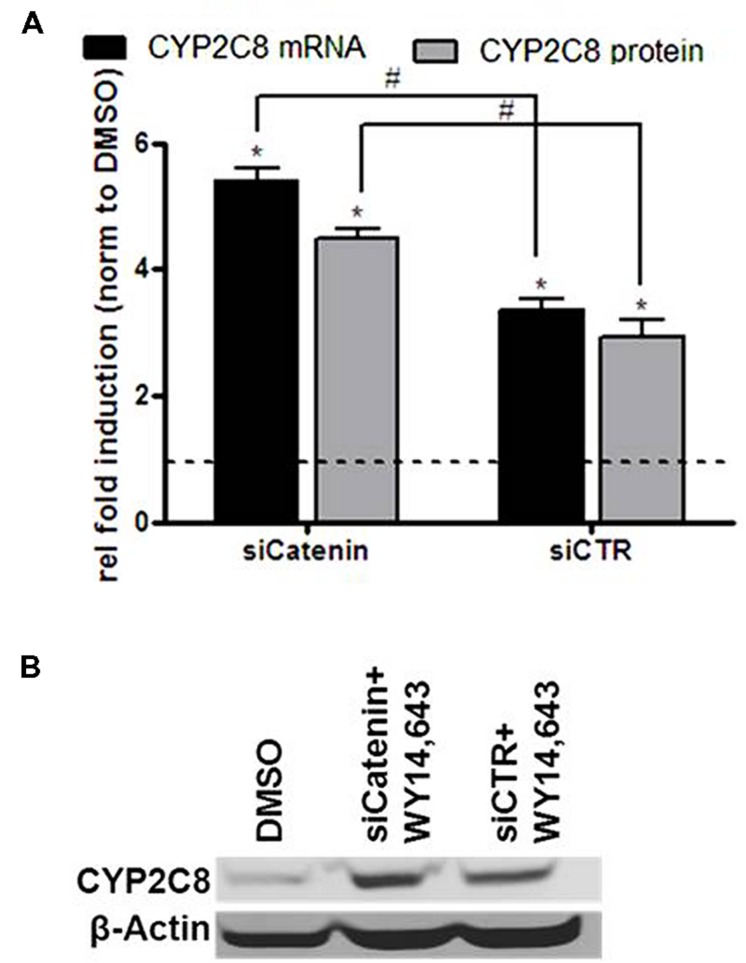
**Inhibitory role of β-catenin in the ligand-mediated induction of CYP2C8 by PPARα. (A)** mRNA (gray bars) and protein (black bars) expression levels of CYP2C8 were analyzed by quantitative real-time qRT-PCR and Western blotting, respectively, following treatment of HepaRG cells with 100 μM of WY14,643 in the presence of β-catenin-targeting siRNA (siCatenin) or non-targeting control (siCTR). Shown are mean values of three independent experiments ±SD. ^∗^, Statistically significant (*P* < 0.05) compared with DMSO treatment set to 1; #, statistically significant (*P* < 0.05) when siCatenin-treated cells compared to control siCTR-treated cells. **(B)** Representative western blot analysis of CYP2C8 protein expression following WY14,643-mediated activation of PPARα in the absence (lane siCatenin + WY14,643) or presence (lane siCTR + WY14,643) of β-catenin.

We recently reported on the crosstalk between the PPARα and WNT/β-catenin pathways in the regulation of P450 enzymes which results in an inhibitory influence of β-catenin on PPARα-mediated induction of CYP3A4 as well as CYP2C8 mRNA (Thomas et al., 2015b). Here we simultaneously analyzed mRNA and protein expression of CYP2C8 following PPARα activation by WY14,643, either in the presence of β-catenin targeting siRNA, siCatenin, or non-targeting siRNA, siCTR (**Figure 6**). In line with our previous observations, knockdown of β-catenin in the presence of WY14,643 led to increased CYP2C8 expression by activated PPARα (**Figure 6A**) on the mRNA (gray bars) and protein (black bars) level (**Figure 6B**). Thus it can be concluded that in HepaRG cells β-catenin exerts a similar inhibitory modulation on PPARα-mediated CYP2C8 induction as previously shown for CYP3A4 regulation.

## Discussion

In the present study, we demonstrated direct binding of the nuclear receptor PPARα to the CYP2C8 promoter resulting in its transcriptional activation and regulation of CYP2C8 expression in hepatocytes and in human liver. We identified two specific regulatory elements that are essential for PPARα-mediated transactivation. One of them corresponds to the previously identified CAR/PXR-binding site at –8806 bp (DR-4) that had been shown to be essential for the activation of the CYP2C8 promoter by both the PXR ligand rifampicin and the human CAR ligand, CITCO (Maglich et al., 2003). In addition we identified a novel PPRE DR-1motif at –2762/–2775 bp region, which is independently of the former site sufficient for PPARα-mediated regulation.

For the direct regulation of CYP2C8 expression by PPARα we provide three lines of evidence: first, ChIP in HepaRG cells revealed a PPARα binding region between –2500 and –3500 bp upstream of transcriptional start site *in vivo*; second, two motifs for PPARα/RXRα binding (DR4-A and DR1-C) were identified and further confirmed by EMSA; and third, reporter gene analysis demonstrated that both of these motifs are essential and sufficient for transcriptional activation by PPARα. The DR4-A motif was previously shown to be a binding site for PXR and CAR. Although EMSA revealed a relatively weak signal of PPARα/RXRα binding to this region, luciferase activity assay further supported the relevance of this site for PPARα-mediated regulation (**Figure 5B**). We hypothesize that probably a multifactorial complex containing PXR/CAR and PPARα is responsible for the transcriptional regulation on this motif. The DR1-C site displayed the strongest binding affinity, in agreement with its strong functional role, as demonstrated by mutational analysis. The presence of several nearby and overlapping transcription factor binding sites may lead to protein–protein interactions that could be explained by the robustness of the regulatory system by several nuclear receptors. In general, our experiments suggest both constitutive and inducible transactivation of CYP2C8 by PPARα. Interestingly, pretreatment of hepatocytes with the CYP2C8 canonical substrate, amodiaquine, further increased enrichment of PPARα within PBR-C as assessed by ChIP assay. Although the exact molecular mechanisms of this increased occupation are currently unclear, it should be noted that WY14,643 did not show such an effect on the CYP3A4 promoter occupancy (Thomas et al., 2013).

For the characterization of genetic and non-genetic factors influencing interindividual variability of hepatic CYP2C8 expression we used our well characterized human liver cohort including 150 white individuals with comprehensive clinical documentation. We observed that environmental and other non-genetic factors have a moderate influence on hepatic CYP2C8 phenotype. Association of CYP2C8 with deregulated bile acid homeostasis and drug induced cholestasis have been reported before (Daly et al., 2007; Rieger et al., 2013). In particular for diclofenac-induced cholestasis, it was suggested that allelic variants in *UGT2B7*, *CYP2C8*, and *MRP2* may cause an increase in the level of reactive metabolites leading to protein-diclofenac adducts that then produce toxicity. The observation that the two linked PPARA variants, rs4253728, and rs4823613, previously shown to influence CYP3A4 expression and function (Klein et al., 2012), also affect expression and activity of CYP2C8 is well explained by the direct transcriptional regulation of CYP2C8 by PPARα and further confirms the major finding of this study. However, whether the influence of these polymorphisms is sufficient to improve *CYP2C8* pharmacogenetic prediction remains to be studied.

In addition to the significance for drug–drug interactions, our findings may have further clinical relevance for the treatment of lipid disorders and plasma dyslipidemia with PPARα ligands. As a variety of dietary and endogenous lipids, including saturated and unsaturated fatty acids, phospholipids, eicosanoids, and many derivatives and metabolites, have been implicated in PPARα activation, our findings suggest an intricate interplay between intermediary metabolism, nutritional status, and biotransformation. Of note, in our previous study we found induction of several P450s including CYP2C8 by the common nutritional phospholipid, POPC (Thomas et al., 2013). The current data on direct P450 induction through PPARα suggests a link between endogenous substances and the regulation of drug biotransformation in human liver and stresses a clear need to readdress the potential for drug–drug interactions, which may depend on nutritional status. This is of particular importance for newly developed PPARα ligands to target obesity, insulin resistance, and diabetes (Lalloyer and Staels, 2010).

## Conclusion

We have elucidated the mechanistic basis for constitutive and inducible transcriptional regulation of CYP2C8 by PPARα. PPARα thus contributes to a transcriptional network so far including CAR, PXR, GR, and HNF4 as direct regulators of CYP2C8 expression, while inducibility is also modulated via WNT/β-catenin pathway. Through these studies, we have identified two specific elements that are essential for transcriptional activation by PPARα, and unraveling this cellular signaling pathway will help to better understand the physiological role of the CYP2Cs and the factors that control their inducibility and contribute to the variability observed in humans.

## Author Contributions

MT, SW, BK, MT (Oulu), and KK structured and conducted the experiments and analyzed the data. MT, MS, and UZ designed the study and wrote the manuscript.

## Conflict of Interest Statement

The authors declare that the research was conducted in the absence of any commercial or financial relationships that could be construed as a potential conflict of interest.

## Footnotes

1 www.r-project.org

2 http://CRAN.R-project.org/package=quantreg

3 http://CRAN.R-project.org/package=RVAideMemoire

## Abbreviations

CAR: constitutive androstane receptor
ChIP: chromatin immunoprecipitation
CYP: cytochrome P450
DMSO: dimethyl sulfoxide
DR: direct repeat
EMSA: electrophoretic mobility shift assays
GGT: serum gamma glutamyl transferase
HMGCR: 3-hydroxy-3-methyl-glutaryl-CoA reductase
HNF: hepatocyte nuclear factor
PBR: PPARa-binding region
P450: cytochrome P450
PPARα: peroxisome proliferator-activated receptor alpha
PPRE: peroxisome proliferator response element
PXR: pregnane X receptor
RXR: retinoid X receptor
siRNA: small interfering RNA
WY14,643: 4-chloro-6-(2,3-xylidino)-2-pyrimidinylthio acetic acid
