# Supplementary material for: Peroxisome proliferator-activated receptor alpha, PPARα, directly regulates transcription of cytochrome P450 CYP2C8
Source: Front Pharmacol. 2015 Nov 4;6:261. doi: 10.3389/fphar.2015.00261 (PMC4631943; doi:10.3389/fphar.2015.00261)
Supplement: Supplementary file 1 [file Table_1.DOCX]

**Peroxisome proliferator-activated receptor alpha, PPARα, directly regulates transcription of cytochrome P450 CYP2C8**

Maria Thomas, Stefan Winter, Britta Klumpp, Miia Turpeinen, Kathrin Klein, Matthias Schwab, Ulrich M. Zanger

**Supplementary Table 1.** List of oligonucleotides used for electromobility shift assays (EMSA). Shown is the sense strand. Two oligonucleotides (Nr. 10, PPRE DR-1 motif and Nr. 13, CAR/PXR DR-4 motif), which revealed a direct binding of PPARa/RXRa heterodimer, are shown in italics; the interspace nucleotides between two direct repeats are shown in bold.

| **Probe no in EMSA** | **Position**  **(bp relative to the CYP2C8 transcription start site )** | **Length**  **(nucleotides)** | **Sequence 5'→3'** |  |  |
| --- | --- | --- | --- | --- | --- |
| PPRE pos. control | -248/-279 of rat ACOX promoter | 31 | CCGCCAAGCTTGCTCCGCCAGGTCACAGGTC | |  |
| 1 | -18/-39 | 22 | AGCTGTGAGCTTGCACTCCAAA | |  |
| 2 | -144/173 | 30 | TTTCTGAGTGGACTTTGGCCCATGGATAGA | | |
| 3 | -316/-338 | 23 | GAATATACTCAAAATTCAATATT | |  |
| 4 | -362/-385 | 24 | ATTTATGACCTTGAGGGAAATCAG | |  |
| 5 | -890/-913 | 24 | ACTCTGGGAGAACAGGACACCTGT | |  |
| 6 | -1897/-1927 | 31 | TTTTAAGCAAAACATCCAGTTAAACCGGGCC | | |
| 7 | -1909/-1933 | 25 | CATCCAGTTAAACCGGGCCACCAAG | |  |
| 8 | -2027/-2051 | 25 | AATGTGGGTCAGACGTGTTTGGTTT | |  |
| 9 | -2040/-2064 | 25 | CGTGTTTGGTTTTATAACCTTTAAA | |  |
| 10 | -2759/-2782 | 24 | *AACCAAAGTTCG****A****AGTTCACATAG* | |  |
| 11 | -2767/-2791 | 25 | TTCGAAGTTCACATAGGGTTTACTG | |  |
| 12 | -4173/-4195 | 23 | AAAACTGGGCAAAGTTCCAATTG | |  |
| 13 | -8802/-8828 | 26 | *ATAAGTCAACT****TTGA****TGACCCCATTT* | |  |
| 14 | -9551/-9582 | 32 | CAGGTGGATCACAAGGTCAGGAGATCGAAACC | | |
| 15 | -9934/-9956 | 23 | GACAGTGGGAGCAGGTCAGTGGG | |  |
